# NBD2 Is Required for the Rescue of Mutant F508del CFTR by a Thiazole-Based Molecule: A Class II Corrector for the Multi-Drug Therapy of Cystic Fibrosis

**DOI:** 10.3390/biom11101417

**Published:** 2021-09-28

**Authors:** Chiara Brandas, Alessandra Ludovico, Alice Parodi, Oscar Moran, Enrico Millo, Elena Cichero, Debora Baroni

**Affiliations:** 1 Istituto di Biofisica, Consiglio Nazionale delle Ricerche, Via De Marini 6, 16149 Genova, Italy; brandas.chiara@hsr.it (C.B.); alessandra.ludovico@ibf.cnr.it (A.L.); oscar.moran@ibf.cnr.it (O.M.); 2 Department of Experimental Medicine, Section of Biochemistry, University of Genova, Viale Benedetto XV 1, 16132 Genova, Italy; alice.parodi1994@gmail.com (A.P.); enrico.millo@unige.it (E.M.); 3 Department of Pharmacy, Section of Medicinal Chemistry, School of Medical and Pharmaceutical Sciences, University of Genova, Viale Benedetto XV 3, 16132 Genova, Italy; cichero@difar.unige.it

**Keywords:** cystic fibrosis transmembrane conductance regulator (CFTR), cystic fibrosis, F508del CFTR, CFTR correctors, CFTR domains, aminoarylthiazoles

## Abstract

Cystic fibrosis (CF) is caused by loss-of-function mutations in the CF transmembrane conductance regulator (CFTR) protein, an anion channel that regulates epithelial surface fluid secretion. The deletion of phenylalanine at position 508 (F508del) is the most common CFTR mutation. F508del CFTR is characterized by folding and trafficking defects, resulting in decreased functional expression of the protein on the plasma membrane. Several classes of small molecules, named correctors, have been developed to rescue defective F508del CFTR. Although individual correctors failed to improve the clinical status of CF patients carrying the F508del mutation, better results were obtained using correctors combinations. These results were obtained according to the premise that the administration of correctors having different sites of action should enhance F508del CFTR rescue. We investigated the putative site of action of an aminoarylthiazole 4-(3-chlorophenyl)-N-(3-(methylthio)phenyl)thiazol-2-amine, named FCG, with proven CFTR corrector activity, and its synergistic effect with the corrector VX809. We found that neither the total expression nor the maturation of WT CFTR transiently expressed in human embryonic kidney 293 cells was influenced by FCG, administrated alone or in combination with VX809. On the contrary, FCG was able to enhance F508del CFTR total expression, and its combination with VX809 provided a further effect, being able to increase not only the total expression but also the maturation of the mutant protein. Analyses on different CFTR domains and groups of domains, heterologously expressed in HEK293 cells, show that NBD2 is necessary for FCG corrector activity. Molecular modelling analyses suggest that FCG interacts with a putative region located into the NBD2, ascribing this molecule to class II correctors. Our study indicates that the continuous development and testing of combinations of correctors targeting different structural and functional defects of mutant CFTR is the best strategy to ensure a valuable therapeutic perspective to a larger cohort of CF patients.

## 1. Introduction

Cystic fibrosis (CF) is an autosomal recessive, lethal disorder caused by loss-of-function mutations in the CF transmembrane conductance regulator (CFTR) protein, a cAMP-dependent anion channel expressed primarily at the plasma membrane of secretory epithelia in the airways, pancreas, intestine and other tissues. In the airways, defective or absent chloride and bicarbonate transport across the epithelium, arising from CFTR mutations causes the accumulation of a sticky and viscous mucus that impedes mucociliary clearance, constitutes the milieu for bacterial colonization and inflammation, and eventually causes severe lung damage and even lung failure [1,2].

CFTR is a multidomain glycoprotein belonging to the ABC transporter super-family formed by five distinct domains: two membrane-spanning domains (MSD1 and MSD2), each having six helices that completely cross the phospholipid bilayer and contribute to form the ion channel, two cytosolic nucleotide binding domains (NBD1 and NBD2), which bind and hydrolyse ATP, and a regulatory domain (R domain), whose phosphorylation is needed for channel gating [1,3]. The structure of wild type (WT) full-length CFTR has been resolved by cryoelectron microscopy [4,5,6,7].

More than 2000 different mutations in the CFTR gene (MIM 602421, http://www.genet.sickkids.on.ca/, accessed on 15 June 2021) have been identified, and more than 300 of them can cause CF (https://cftr2.org/, accessed on 15 June 2021). CFTR mutations are divided into six classes according to the degree of CF disease severity and the mechanism that disrupts CFTR function [8,9]. The deletion of phenylalanine at position 508 (F508del) is the most common CFTR mutation, being present in at least 90% of CF patients. It primarily abolishes the CFTR protein’s ability to mature and to traffic to the plasma membrane. As a result, the mutant protein is detected by cell quality control and degraded by the ubiquitin/proteasome system [10,11,12,13]. However, the few mutant channels that succeed in reaching the plasma membrane possess additional defects, consisting in reduced open channel probability [14,15,16] and a shorter half-life with respect to WT CFTR [9,12,17,18].

Since it was discovered that incubation at low temperature, or treatment with high concentrations of chemical chaperones such as glycerol [19,20], partially restores F508del CFTR trafficking in vitro, an intensive effort has been put into high-throughput screening aimed to identify molecules, named correctors, able to rescue F508del CFTR to the plasma membrane [21,22]. Among discovered compounds, lumacaftor (VX809) represents the first corrector molecule to be tested in CF patients homozygous for the F508del mutation. Despite impressive rescue levels of F508del CFTR in patient-derived primary cultures of human bronchial epithelial cells, VX809, as well as newer derivatives (i.e., Tezacaftor, VX661), showed only modest efficacy in improving lung function of CF patients [23,24], probably because their action is limited to only one of the multiple defects of mutant F508del CFTR [25,26]. It is now widely accepted that administration of combinations of correctors that act either simultaneously or sequentially at multiple sites of the CFTR folding pathway could have a synergistic effect in the rescue of F508del CFTR expression [27,28,29,30,31].

The newly developed CFTR corrector elexacaftor (VX445) in the combination named trikafta, where it is combined with the corrector VX661 and the potentiator ivacaftor (VX770), administered to F508del-homozygous and F508del-heterozygous with minimal function mutation patients, has been shown to be effective and safe, and results in a clinical response that is appreciably better than that of previous CFTR modulators. In fact, in these patient cohorts, the triple-combination regimens significantly reduced sweat chloride concentration, decreased the incidence of pulmonary exacerbations, and ameliorated FEV_1_ [32]. The use of the triple combination (VX445 + VX661 + VX770) gave also positive outcomes in rescuing some other CFTR rare mutations [33,34].

To facilitate the choice of corrector combinations with complementary mechanisms of action, or targeting different defects in the mutant CFTR structure, correctors have been classified into three classes according to their target sites on the CFTR molecule. Class I correctors stabilize NBD1-TMD1 or NBD1-TMD2 interfaces, class II correctors stabilize NBD2 and its interfaces with other CFTR domains, and class III correctors directly stabilize NBD1 [27]. Examples of correctors of class I are VX-809 and 6258, while corr-4a and 3151 belong to class II, and [2-(5-bromo-1H-indol-1-yl)acetic acid (BIA29, a structural analog of BIA) and corrector 3158 belong to class III [35].

In this work, we explored the mechanism of action of FCG, an aminoarylthiazole (AAT) derivative with an already proven efficacy and synergy with VX809, in the rescue of the whole F508del CFTR molecule [36], analysing its effect on different CFTR domains and groups of domains of the CFTR protein. Achieved results were compared with those obtained after treatment with the corrector VX809, administrated either singly or in combination with FCG. Our results confirm that FCG can increase the expression of F508del CFTR heterologously expressed in highly transfectable human embryonic kidney 293 (HEK-t) cells, and that this effect is synergistic with that exerted by VX809. On the contrary, both compounds did not exert any effect on either total expression or maturation of WT CFTR transfected in HEK-t cells.

Furthermore, our biochemical analysis identified NBD2 as the CFTR domain mainly involved in the rescue action achieved by FCG. In fact, this molecule demonstrated the ability to increase not only the expression but also the stability of NBD2 heterologously expressed in HEK-t cells. Computational investigation of FCG-NBD2 interaction reinforced these findings, exploring for FGC the putative bioactive conformation at the NBD2 domain as well as identifying the most probable binding cavity at the aforementioned NBD2 domain. This information will allow us to enrich the available structure-based information for the further development of thiazole-containing derivatives targeting NBD2, as promising CFTR modulators, to be exploited in combination with VX809 or class I correctors [27]. As already highlighted in previous studies [37,38,39,40], VX809 enhanced the expression of MSD1 and all the constructs where this domain was contained (CFTR N-half, ΔNBD2, a construct of CFTR lacking the NBD2, and the F508del CFTR whole molecule). In contrast, it had no effect on the expression of all the constructs where the NBD2 was expressed.

Our results confirm that the administration of a combination of drugs targeting different CFTR domains responsible for different structural and functional defects of the mutant protein is the best strategy to achieve significant clinical outcomes in the cure of CF. Finally, our analyses highlight, once more, that the family of AATs to which FCG belongs represents a privileged scaffold whose properties deserve to be further investigated for the rational design of novel and more effective derivatives for the treatment of CF.

## 2. Materials and Methods

### 2.1. Chemicals

The 4-(3-chlorophenyl)-N-(3-(methylthio)phenyl)thiazol-2-amine (FCG) compound was synthesized as already described [36]. Lumacaftor (VX809) was purchased from Selleck Chemicals (Munich, Germany). If not explicitly indicated in the text, all other chemicals and culture media components were provided by Sigma-Aldrich (Milan, Italy).

### 2.2. Cell Culture Conditions and Evaluation of Compound Toxicity

Human, highly transfectable embryonic kidney 293 (HEK-t) cells were purchased from the Interlab Cell Line Collection (Genoa, Italy). Cells were grown in Dulbecco’s modified Eagle’s medium (DMEM) supplemented with 2 mM l-glutamine, 1% penicillin-streptomycin (100 U/mL) and 20% fetal bovine serum (FBS) at 37 °C and 5% CO_2_. To prevent the loss of differentiation potential, cells were not allowed to become confluent. In these cells, FCG and VX809 toxicity was evaluated by the trypan blue exclusion staining method [37,41]. HEK-t were exposed to the compounds for 24 h at the following concentrations: 50, 32, 16, 8, 4, 2, 1, 0.5, 0.25, 0.125 and 0 (vehicle, DMSO) μM. After exposition, cells were trypsinized and harvested for toxicity evaluation. To avoid an underestimation of the number of dead cells, the cells that were detached from the plates during exposure to the compounds were collected and considered. For each condition tested, at least four biological replicates were evaluated. In each experiment at least 100 cells were considered. The concentration resulting in half maximum toxicity, TD_50_, was calculated by plotting the percentage of cell survival against FGC concentration (*C*), and fitting the data according to:

(1) $$\%survival=100*TD_{50}/\left(TD_{50}+C\right)$$

### 2.3. Generation and Expression of CFTR Constructs

Plasmids codifying for whole CFTR molecule (residues 1–1480), as well as those encoding MSD1 (M1, residues 1–388M), NBD1 (N1, residues 348–633), MSD2 (M2, residues 837–1218), NBD2 (N2, residues 1210–1480), CFTR N-half (M1N1, residues 1–633), CFTR C-half (M2N2, residues 837–1480), and CFTR lacking the NBD2 domain (∆NBD2, residues 1–1172) were subcloned between Hind III and XhoI, while the construct codifying for the R domain (R, residues 645–834) between the Hind III and EcoRI restriction sites of the expression vector pCDNA3 (Invitrogen, Paisley, UK) [36]. The cDNAs encompassing *phenylalanine at position 508 of the CFTR molecule* were further modified by site-directed mutagenesis to introduce the F508del deletion, using a QuickChange kit (Stratagene, Santa Clara, CA, USA). The mutation was verified by sequencing (Biofab Research, Rome, Italy). A scheme of the constructs used in this work is shown in Figure S1.

For transfection, HEK-t cells were plated onto poly-l-lysine-coated culture dishes and grown to 65% confluence in a complete medium. Cells were transiently transfected using Lipofectamine 2000 (Invitrogen, Paisley, UK) with 4 μg of cDNA. The transfection medium (DMEM supplemented with 2 mM l-glutamine and without FBS) was replaced after 6 h with a fresh complete medium containing 10 µM FGC, 5 µM VX809, or 10 µM FGC+ 5 µM VX809 or vehicle DMSO (control). Cells were harvested after 24 h.

### 2.4. RNA Isolation, Reverse Transcription, and Quantitative Real-Time Polymerase Chain Reaction

Total RNA was isolated using the RNeasy Mini kit (Qiagen, Hilden, Germany), and first-strand cDNA was synthesized from 2 µg of RNA using the RevertAid First Strand cDNA Synthesis Kit and random hexamers according to the manufacturer’s instructions (Fermentas, Burlington, ON, Canada). First-strand cDNA from transfected HEK-t cells was employed as the template in a quantitative real-time polymerase chain reaction (qRT-PCR) in a CFX Connect Real-Time PCR Detection System instrument (Bio-Rad Laboratories, Hercules, CA, USA). The sequences of the oligonucleotide primer pair specific for the full-length CFTR, M1, N1, R, M2, N2, M1N1, M2N2 and ∆NBD2, for glyceraldehyde-3-phosphate-dehydrogenase (GAPDH) used as housekeeper gene, and the amplification conditions, are listed in Table S1. Changes in cDNA amount were evaluated using the comparative cycle threshold (*C_t_*) method. Each sample was run in at least triplicate.

### 2.5. Western Blot

Cells were lysed in a RIPA lysis buffer (50 mM Tris-HCl, pH 8.0, 150 mM NaCl, 1% Triton X-100, 1% sodium deoxycholate, 0.1% SDS) containing a complete protease inhibitor cocktail (Sigma-Aldrich). Protein concentration was determined by Bradford’s method using bovine serum albumin as the standard. Equal amounts of proteins (30 μg) were subjected to SDS-PAGE and transferred to a PVDF membrane (Millipore, Billerica, MA, USA). Blots were incubated with anti-CFTR monoclonal primary antibodies raised against the N-terminus (clone MM13-4, Millipore, dilution 1:200), the C-terminus (clone M3A7, Millipore, dilution 1:200), the NBD1 (clone L12B4, Millipore, dilution 1:200), the R domain (clone 13-1, Novus Biologicals, dilution 1:100), and with a rabbit polyclonal primary antibody raised against residues 1150–1200 of the CFTR protein, to detect full-length CFTR, M1, M1N1, ∆NBD2, N2, M2N2, R and M2 domains, respectively. Goat anti-mouse or anti-rabbit horseradish peroxidase conjugated antibodies (dilution 1:2000; Santa Cruz Biotechnologies, Dallas, TX, USA) were used as secondary antibodies. Immunodetection was performed using Amersham ECL PLUS detection reagents (GE Healthcare Europe GmbH, Milan, Italy), and the images were captured by using Amersham Hyperfilm ECL. To confirm the homogeneity of the loaded proteins, immunoblots were stripped by incubating them with stripping buffer (62.5 mM Tris-HCl, pH 6.8, 10% SDS and 1% β-mercaptoethanol) for 30 min at 55 °C and reprobed with an antiactin polyclonal antibody (1:2000, Sigma). Untransfected cell lysates, used as negative controls, were assayed with anti-CFTR and antiactin antibodies. For quantification, densitometry of the Western blot images was done with ImageJ software (U.S. National Institutes of Health, Bethesda, MD, USA). For each lane, the bands, analysed as regions of interest, were quantified and normalized to the intensity of the band corresponding to the actin detected in the stripped PVDF membranes. The Western blot of each analysed condition was repeated at least in four independent experiments.

### 2.6. Cycloheximide Chase Assay

To evaluate the stability of the NBD2 polypeptide, the HEK-t cells were transfected with the plasmid containing the cDNA encoding this construct and incubated for 18 h in the presence of DMSO (control), 10 μM FGC, 5 μM VX809 or 10 μM FGC + 5 μM VX809. Protein synthesis was then inhibited by addition of 0.5 mg/mL cycloheximide. Cells were harvested at six different time points (after 0, 1, 2, 4, 6 and 8 h), and samples of whole cell SDS extracts were subjected to immunoblot analysis with monoclonal antibody MM13-4 raised against the N-terminus of the CFTR protein.

### 2.7. Statistics

Data were analysed using Igor Pro software (version 8.0.3.3, Wavemetrics, Lake Oswego, OR, USA). Results are expressed as mean ± SEM (standard error of the mean). A post hoc multiple comparison Dunnett’s test was run after a significant one-way analysis of variance (ANOVA) to compare data sets. In all cases, significance was accepted for a probability of *p* < 0.05.

### 2.8. Molecular Modelling Studies

All the studied correctors were manually built by the MOE Buildermodule implemented in the MOE program and were then parametrized (AM1 partial charges as calculation method) and energy minimized by the Energy Minimize tool of the same software, using MMFF94x forcefield and an RMS (root mean square) equal to 0.0001 Kcal/mol/A^2^, to produce a single low-energy conformation for each ligand [42].

Docking calculations were performed considering the X-ray crystallographic data of the NBD2 domain of the CFTR protein downloaded from the protein data bank (PDB code 6UK1; resolution 2.69 Å) [43]. The most probable binding sites within the biological target were detected by means of MOE Site Finder, choosing the best top ranked cavity (namely BS1), using a protocol detailed elsewhere [44]. Finally, all the collected sites based on hydrophilic/hydrophobic properties were ranked according to their Propensity for Ligand Binding (PLB) score, which is based on the amino acid composition of the pocket as described in the literature [45].

The following docking calculations were performed by means of the DOCK tool implemented in MOE, choosing as the binding site the previously mentioned BS1, according to a docking protocol previously described [46,47]. Briefly, the alpha triangle as the placement algorithm was selected, running by superposition of ligand atom triplets and triplets of receptor site points. The receptor site points were represented by alpha sphere centers. At each iteration, a random conformation was selected. A random triplet of ligand atoms and a random triplet of alpha sphere centers were used to determine the pose. Calculation of the enthalpy-based Affinity dG scoring function allowed scoring of the fifty poses generated, while the Induced Fit method was exploited to refine the previous poses to the final ten docking poses. These were rescored based on Alpha HB methodology based on H-bonding estimation.

This Affinity dG function estimates the enthalpic contribution to the free energy of binding using a linear function:

(2) $$G=C_{hb}f_{hb}+C_{ion}f_{ion}+C_{hmlig}f_{mlig}+C_{hh}f_{hh}+C_{hp}f_{hp}+C_{aa}f_{aa}$$

where the *f* terms fractionally count atomic contacts of specific types and the *C* terms are coefficients that weight the term contributions to the affinity estimate. The individual *hb* terms represent interactions between hydrogen bond donor-acceptor pairs. An optimistic view is taken in that two hydroxyl groups are assumed to interact in the most favourable way by *ion* ionic interactions. A Coulomb-like term is used to evaluate the interactions between charged groups. This can contribute to, or detract from, binding affinity *mlig* via metal ligation. Interactions between nitrogens/sulfurs and transition metals are assumed to be metal ligation interactions, i.e., *hh*: hydrophobic interactions, for example, between alkane carbons. These interactions are generally favourable *hp*: interactions between hydrophobic and polar atoms. These interactions are generally unfavourable, as in *aa*, an interaction between any two atoms. This interaction is weak and generally favourable.

An induced Fit approach allows maintenance of flexible protein sidechains within the selected binding site, which are to be included in the refinement stage. The derived docking poses were prioritized by the score values of the lowest energy pose of the compounds docked to the protein structure, as follows: S is the final score, which is the score of the last stage of refinement and *E_conf* is the energy of the conformer. If there is a refinement stage, this is the energy calculated at the end of the refinement. *E_place* is the score from the placement stage, the E_score1 and E_score2 score from rescoring stages 1 and 2, and the *E_refine:* score from the refinement stage, calculated to be the sum of the van der Waals electrostatics and solvation energies, under the Generalized Born solvation model (GB/VI).

### 2.9. In Silico Evaluation ADME C of FCG

The prediction of descriptors explaining ADME properties was developed by means of the Advanced Chemistry Development (ACD) Percepta platform [43] based on training libraries implemented in the software, which refer to different series of derivatives whose pharmacokinetic properties have been experimentally investigated.

## 3. Results

### 3.1. Cytotoxicity of FCG and VX809

When administrated individually, FCG and VX809 exerted comparable toxic effects on HEK-t cells, as evaluated by the trypan blue exclusion test. Indeed, their TD_50_ values were 52.8 ± 0.8 μM, and 55.1 ± 0.5 μM, respectively. Cell viability of HEK-t cells after 24 h exposure to different concentrations of FCG and VX809 is shown in Table S2. Due to their relative low cytotoxicity, we chose to use FCG and VX809 at 10 and 5 μM, respectively, which are the concentrations that yielded more than 85% of cell survival, to perform further experiments. The estimated half effective concentration of FCG was 5.3 μM [36].

### 3.2. Effect of Compounds on the Expression of the mRNAs Codifying CFTR Whole Molecule, Single and Groups of Domains

The expression levels of the mRNA coding for full-length WT and F508del CFTR constructs in transfected HEK-t cells, evaluated by qRT-PCR, were not statistically different. Interestingly, treatment of cells with FCG, VX809, and FCG + VX809 did not modify the expression of the CFTR mRNA either in WT CFTR or in F508del CFTR-transfected cells. Data are presented in Table S3.

None of the examined compounds altered the relative abundance of M1. Analogously, treatment with FCG, VX809 and FCG + VX809 did not change the expression level of WT or F508del N1, N2, R and M2 (Table S4).

The relative abundance of WT M1N1 mRNA in untreated transfected cells was not changed upon treatment with FCG, VX809, and FCG + VX809. Similarly, the F508del M1N1 mRNA yield in untreated cells was not modified by the treatment with the compounds under study. Data are presented in Table S5. Analogously, treatment with correctors did not change the expression level of the M2N2 mRNA in transiently transfected HEK-t cells. Finally, the amount of the mRNA extracted from cells transfected with WT or F508del ∆NBD2 was similar in untreated cells and in cells treated with FCG, VX809, and FCG + VX809 (Table S5).

In untransfected HEK-t cells, mRNA codifying for the full-length CFTR, M1, N1, R, M2, M1N1, M2N2, and ∆NBD2 was not detected (data not shown). From the obtained results, we concluded that none of the compounds under study showed an effect on the transcription of the cDNA encoding whole CFTR protein or any single domain or group of CFTR domains.

### 3.3. Effects of Compounds on the Expression of Full-Length WT and F508del CFTR Proteins

**Figure 1:**
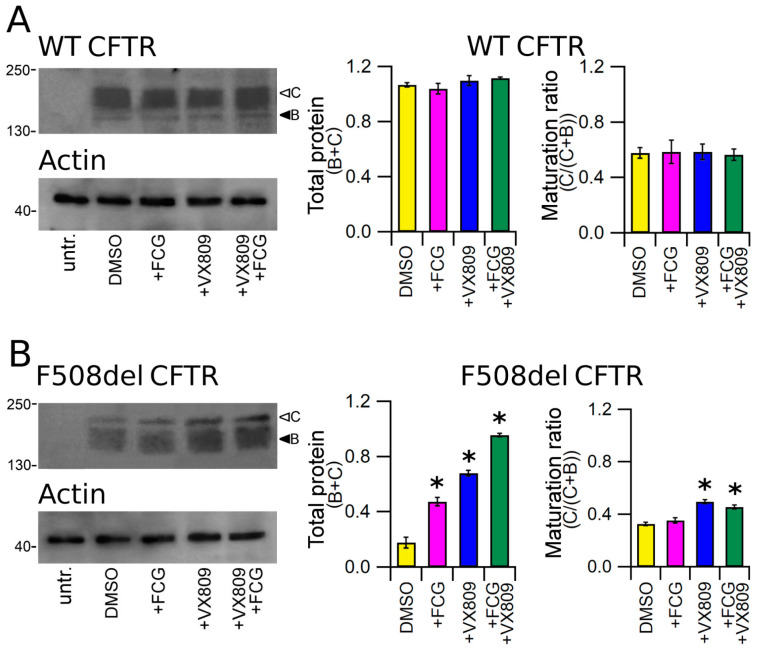
Detection of full-length CFTR proteins by Western blot. Detection of WT (**A**) and F508del CFTR (**B**) in lysates of untransfected and transiently transfected HEK-t cells treated with DMSO (control), FCG, VX809, and FCG + VX809 is shown in the upper panels on the left. Expression of the housekeeper protein actin in the same samples is shown in the lower panels. The molecular weight of the proteins of the molecular weight marker that was run in the SDS-PAGE is indicated on the left of each blot. White and black arrowheads indicate the position of bands B and C, respectively. Bar graphs in the middle show the quantification of total protein expression, calculated as the sum of bands B and C. Bar graphs on the right indicate the quantification of the mature, fully glycosylated fraction of the CFTR protein, expressed as C/(C + B) ratio. The expression level of each band was normalized to the level of actin detected in the same samples. Data are expressed as mean ± standard error of the mean (sem) of at least four independent experiments. Statistical comparison of the data was made by Dunnett’s multiple comparison test (all groups against control group). Asterisks indicate a statistical significance versus control: * *p* < 0.05.

To test the effect of the compounds under study on protein expression, we treated HEK-t cells transiently transfected with whole length WT and F508del CFTR for 24 h with 10 μM FCG, 5 μM VX809, 10 μM FCG + 5 μM VX809, or with DMSO as vehicle control. The immunoblot analysis of retrieved whole cell extracts is presented in the upper panels of Figure 1A,B. Both CFTR isoforms were detected by the monoclonal antibody MM13-4 raised against the N-terminal of the CFTR protein as two electrophoretic bands, named B and C, of approximately 160 and 180 kDa, which correspond to the core-glycosylated and the mature, fully processed CFTR, respectively. As expected, the prevalent band in WT CFTR transfected HEK-t cell lysates was the C band (the first lane of the upper panel of Figure 1A). Lysates of cells expressing the F508del CFTR showed a more intense B band, which is consistent with the severe folding and trafficking defects caused by the mutation (first lane of the upper panel of Figure 1B). Neither WT nor F508del CFTR were detected in untransfected HEK-t cells. As shown by the bar graphs of Figure 1, treatment of full-length WT CFTR-transfected cells with FCG, VX809 and FCG + VX809 did not change either WT CFTR total expression (C + B bands) or its maturation ratio, which was expressed as the ratio between the expression of the mature, fully glycosylated protein (band C) and the total CFTR protein (B + C bands). Administration of FCG, VX809 and FCG + VX809 to F508del CFTR-transfected cells significantly enhanced the total expression (B + C bands) of the mutant CFTR protein. The order of compound efficacy in promoting the augment of F508del CFTR total expression was FCG + VX809 > VX809 > FCG. Analogously, treatment with VX809 and FGC + VX809 promoted maturation of the mature, fully glycosylated form of F508del CFTR (C/(C + B)), while treatment with 10 µM FCG alone did not modify the maturation ratio of F508del CFTR.

### 3.4. Effect of Compounds on the Expression of CFTR Single Domains

**Figure 2:**
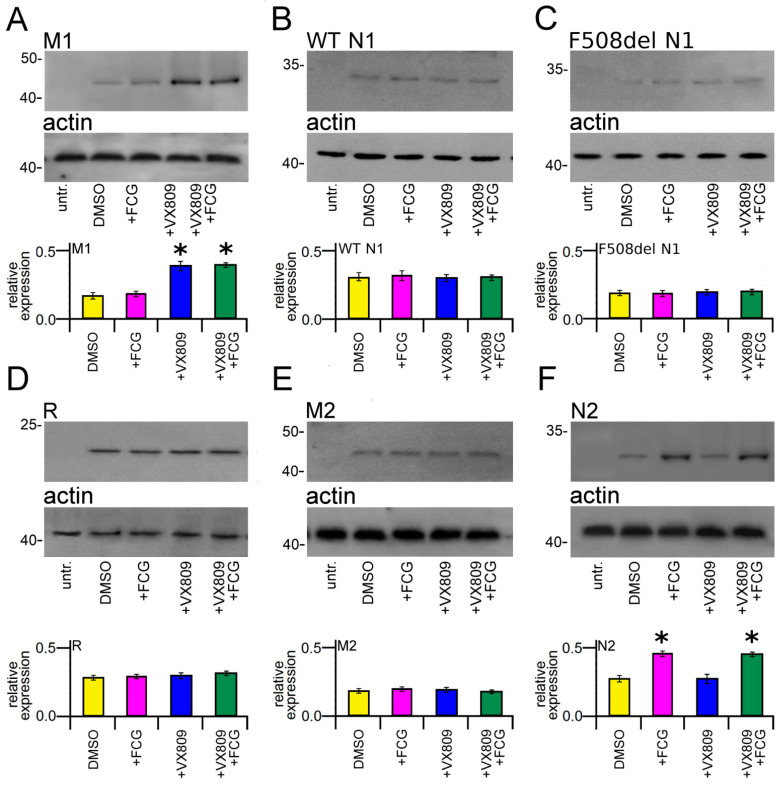
Effect of FCG, VX809, and FCG + VX809 on the expression of CFTR single domains. Western blots of M1 (**A**), WT (**B**) and del508F N1 (**C**), R (**D**), M2 (**E**) and N2 (**F**) CFTR domains in untransfected and in transiently transfected in HEK-t cells treated with DMSO (control) or with 10 µM FCG, 5 µM VX809, and 10 µM FCG + 5 µM VX809, respectively. In the lower blots is shown the expression of actin, used as housekeeping protein. The molecular weight of the proteins of the molecular weight marker that was run in the SDS-PAGE is indicated on the left of each blot. The bar graphs on the bottom of each panel indicate the normalized expression level of each single domain. Data are expressed as mean ± standard error of the mean (sem) of at least four independent experiments. Dunnett’s test was used for data comparison. Asterisks indicate a statistical significance versus control: * *p* < 0.05.

To verify whether the treatment with the compounds under study could determine an increase in the expression of any single domain of the CFTR protein, we transiently transfected HEK-t cells with M1, WT and F508del N1, R, M2 and N2 domains and successively treated them with 10 μM FCG, 5 μM VX809 and 10 μM FCG + 5 μM VX809 for 24 h. Immunoblots of whole cell lysates from CFTR single domains transfected HEK-t cells are shown in the upper panels of Figure 2A–F. The M1 domain was revealed by the MM13-4 antibody as an electrophoretic band of ~45 kDa, while both WT and F508del N1 polypeptides were detected as bands of ~32 kDa by the L12B4 antibody. The R domain has an apparent molecular weight of ~22 kDa. The primary antibodies raised against the M2 and N2 domains revealed these polypeptides as bands of ~47 and ~30 kDa, respectively. Controls in untransfected HEK-t cells showed that no CFTR single domain was detected in the blots by the primary antibodies that were used.

Treatment with FCG did not modify the expression of the M1 domain. On the contrary, the expression of this domain was significantly increased in M1-transfected HEK-t cells treated with VX809 or FCG + VX809 (Figure 2A). Both treatments demonstrated an increasing of the expression of M1 of 2.18 and 2.13-fold with respect to control DMSO treated cells. Notice that the addition of FCG did not increase the VX809 correction.

No significant increase in N1 protein expression was observed in WT and F508del N1-transfected cells upon treatment with FCG or VX809, administrated individually or in combination (Figure 2B,C, respectively). Similarly, these compounds did not produce any significant effect on the expression level of R (Figure 2D) and M2 (Figure 2E) domains. In contrast, the expression of N2 was significantly increased in transfected HEK-t cells treated with FCG and FCG + VX809. In both cases, N2 protein expression similarly increased 1.61-fold with respect to that of control DMSO-treated cells. Treatment with VX809 alone did not modify the expression level of the N2 domain (Figure 2F).

### 3.5. Effect of Compounds on the Expression of CFTR Groups of Domains

**Figure 3:**
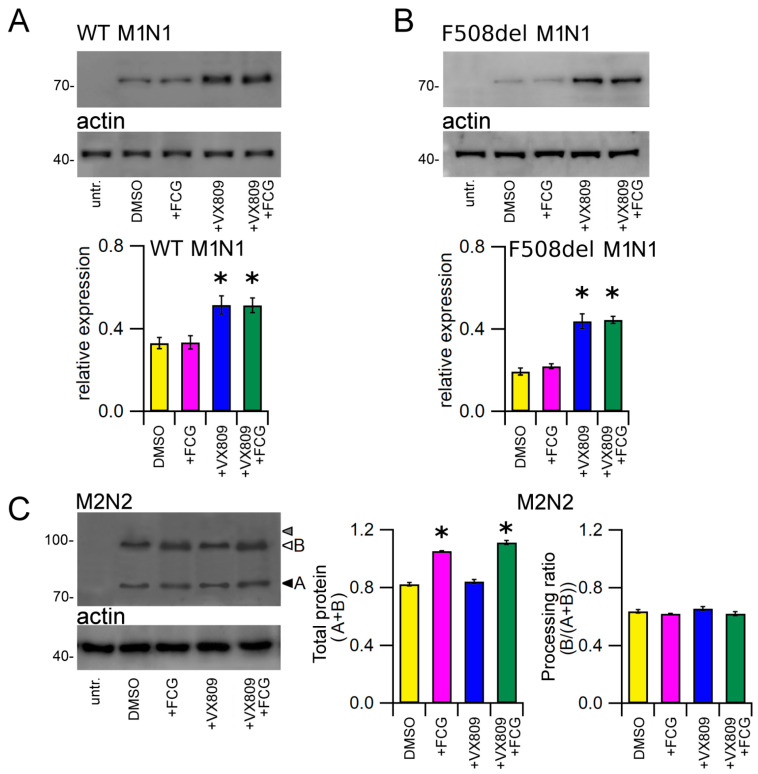
Effect of FCG, VX809, and FCG + VX809 on the expression of CFTR N- and C-halves. Western blots of WT (**A**), F508del M1N1 (**B**) and M2N2 (**C**) in untransfected and in transiently transfected in HEK-t cells treated with DMSO (control) or 10 µM FCG, 5 µM VX809, and 10 µM FCG + 5 µM VX809. White and black arrowheads in (**C**) indicate the position of bands B and A of the M2N2 polypeptide, while grey head arrow indicates the lack of band C, respectively. In the lower blots, the expression of actin, used as housekeeping protein, is shown. The molecular weight of the proteins of the molecular weight marker that was run in the SDS-PAGE is indicated on the left of each blot. The bar graphs on the bottom indicate the normalized expression level of CFTR N- and C-halves. Data are expressed as mean ± standard error of the mean (SEM) of at least four independent experiments. Dunnett’s test was used for data comparison. Asterisks indicate a statistical significance versus the control: * *p* < 0.05.

The lower panels of Figure 3A,B show the Western blot images of WT and F508del M1N1 SDS-PAGE whole cell lysate samples. WT and F508del M1N1 proteins were revealed as electrophoretic bands of ~72 KDa, as expected from the predicted size of the CFTR N-half. In accordance with the sequence analysis, which did not predict any glycosylation site, WT and F508del M1N1 molecules were not glycosylated. WT and F508del M1N1 isoforms were not revealed in untransfected HEK-t whole cell lysates. The quantification of band intensity displayed in the bottom panels of Figure 3A,B shows that the expression levels of the WT and F508del proteins were quite different, being WT M1N1 1.7-fold more expressed than F508del. As expected, the treatment with FCG did not modify the expression of either WT or F508del M1N1 proteins while, different from observations with full-length CFTR isoforms, the administration of VX809 and FCG + VX809 rescued not only the expression of the F508del but also of the WT CFTR N-half proteins. The enhancement of M1N1 protein expression level was equivalent when VX809 was administrated alone or in combination with FCG.

Whole cell extracts from M2N2-transfected HEK-t cells were used to perform an immunoblot analysis aimed at evaluating the expression of the M2N2 protein. In agreement with previous reports showing that CFTR C-half expresses the fully glycosylated form only when it is coexpressed with CFTR N-half [37,38,39], we also found that the M2N2 protein did not express the C band corresponding to the mature fully glycosylated form of the protein (whose position on the blot is shown by the grey head arrow in the upper panel of Figure 3C). On the contrary, SDS-PAGE analysis of M2N2-transfected HEK-t cell samples yielded two bands of lower apparent molecular weight, corresponding to the core-glycosylated (~92 kDa, band B) and, probably, to the unglycosylated (~86 kDa, band A) M2N2 polypeptide (white and black head arrows in the upper panel of Figure 3C). The M3A7antibody raised against the C-terminus of the CFTR did not detect the M2N2 polypeptide in untransfected HEK-t cells. In M2N2 whole cell lysates, the treatment with FCG and FCG + VX809 similarly increased the total expression (A + B band) level of M2N2 polypeptide. In contrast, VX809 alone did not show any significant modification of this parameter. Both FCG and VX809, administrated individually or in combination, did not increase the processing ratio (B/(A + B)) of M2N2 polypeptide, as shown by the bar graphs of Figure 3C.

**Figure 4:**
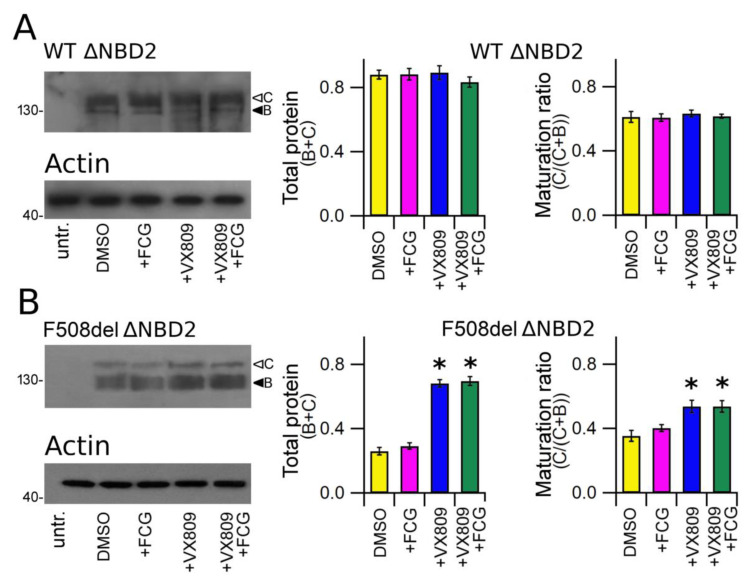
Biochemical analysis of WT and F508del ΔNBD2 expression pattern. (**A**) Electrophoretic mobility of WT ΔNBD2 in untransfected and in HEK-t cells treated with DMSO (vehicle control) or with FCG, VX809 and FCG + VX809, respectively. (**B**) Western blot of whole cell lysates of F508del ΔNBD2 untransfected and F508del ΔNBD2 transfected cells after 24 h of treatment with the above-mentioned compounds. Arrowheads indicate the fully glycosylated (band C) and the core-glycosylated (band B) forms of the ΔNBD2 proteins, respectively. The blots in the bottom show the expression of actin, used as housekeeping protein. The molecular weight of the proteins of the molecular weight marker that was run in the SDS-PAGE is indicated on the left of each blot. Bar graphs in the middle show the quantification of the total ΔNBD2 protein expression, calculated as the sum of bands B and C. Bar graphs on the right indicate the quantification of the mature, fully glycosylated fraction of the ΔNBD2 protein, expressed as C/(C + B) band ratio. Data are expressed as means ± SEM of at least four independent experiments. Statistical significance was tested by Dunnett’s multiple comparisons test (all groups against the control group). Asterisks indicate statistical significance versus DMSO: * *p* < 0.05.

Finally, we tested the effect of the compounds under study on the expression of WT and F508del CFTR that lacked NBD2. Analogously to CFTR, this polypeptide was detected in whole cell lysates of transfected HEK-t cells as two electrophoretic bands of ~130 KDa (B band, immature, partially glycosylated) and ~150 KDa (C band, mature, fully glycosylated) (upper panels of Figure 4A,B). In contrast, it was not recognized in untransfected HEK-t cells (first lanes of the upper panels of Figure 4A,B). The bar graph of Figure 4A shows that neither FGC nor VX809, alone or in combination, were able to enhance WT ∆NBD2 total expression (B + C band) or to increase the expression of its mature, fully glycosylated form (C/(C + B)). Conversely, in lysates from F508del ∆NBD2 HEK-t transfected cells, the treatment with VX809 and FCG + VX809 caused a similar increase of either total protein (C + B band) and mature protein (C/(C + B) band ratio) fractions with respect to control, untreated cells. In F508del ∆NBD2 preparations, when administrated alone, FCG did not augment of the expression level of the total (C + B band) as well as the mature (C/(C + B) band ratio) fractions of the ∆NBD2 protein.

### 3.6. Effect of FCG and VX809 on the Stability of NBD2

**Figure 5:**
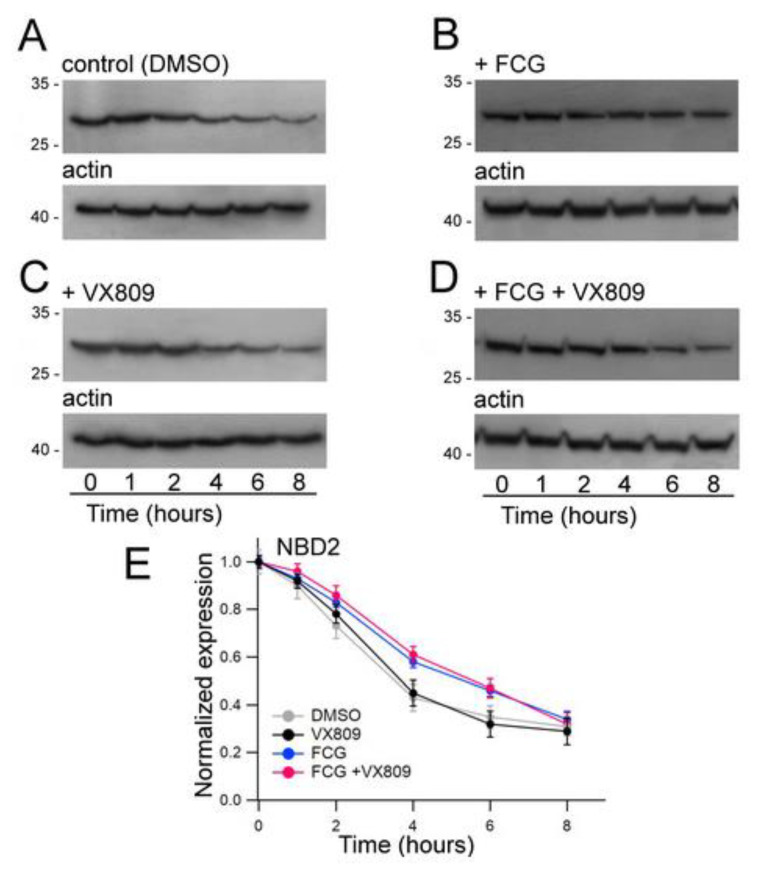
Evaluation of N2 stability by means of the cycloheximide chase approach. Expression of the N2 polypeptide in HEK-t cell lysates treated with DMSO (**A**), 10 µM FCG (**B**), 5 µM VX809 (**C**), 10 µM FCG + 5 µM VX809 (**D**) and subjected to protein synthesis inhibition by means of incubation with 0.5 mg/mL cycloheximide. The lanes of each blot represent six different time points as indicated at the bottom of the figure. For each condition, the expression of the protein actin is shown in the lower panels of (**A**–**D**), respectively. (**E**) Expression of the N2 protein at each time point. Data are ex-pressed as means ± SEM of at least four independent experiments. In the legend of the figure are indicated the symbols used to represent each compound used in the experiment. For all conditions under analysis, the amount of the N2 protein was normalized to actin and expressed relative to time 0.

As FCG seems to mostly affect the expression of NBD2, we assessed whether this compound could exert an effect on the stability of this polypeptide. Furthermore, the impact of the treatment with VX809, FCG + VX809 and DMSO on NBD2 polypeptide half-life was assayed (Figure 5). Cycloheximide chase experiments showed that the expression of N2 in control DMSO untreated samples decayed to 43% after 4 h and to 35% after 6 h from the beginning of the treatment with cycloheximide (Figure 5A). Analogously, after 4 and 6 h from the blockade of protein synthesis with cycloheximide, the expression level of N2 treated with VX809 was 45% and 32% of initial expression, respectively (Figure 5B). Treatment with FCG, administrated alone or in combination with VX809, caused a significant increase of N2 stability. In fact, in FCG and FCG + VX809-treated samples the expression level of N2 resulted in 58% and 61%, and in 46% and the 47%, of initial expressions after 4 and 6 h from the beginning of the treatment with cycloheximide, respectively (Figure 5C,D). Table S6 shows, for each time interval depicted in Figure 5, the values of N2 expression normalized to the intensity of the housekeeper protein actin, and of N2 expression at the beginning of the treatment with cycloheximide. For each condition, the statistical significance, as assayed by Dunnett’s multiple comparisons test (all groups against the control group) is also shown.

### 3.7. Molecular Docking Studies at the NBD2 Domain

We explored the putative binding mode of the FCG corrector by performing molecular docking calculations at the crystallographic structure of the CFTR NBD2 domain [43]. First, we identified the putative binding site (namely BS1) using the MOE Site Finder module, as described in the experimental section [42,43,44,45,46]. Successively, all the molecular docking studies were focused to the BS1 site.

**Figure 6:**
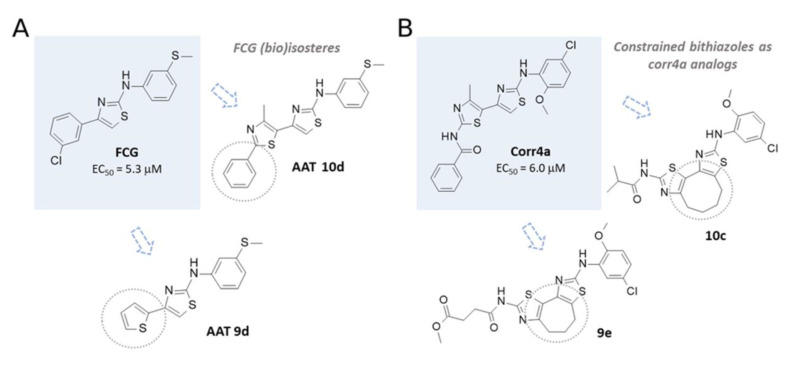
(**A**) Chemical structure of the AAT FCG and related analogues **10d** and **9d**. (**B**) The known F508del CFTR correctors **corr4a** and constrained bithiazoles **9e** and **10c**.

In order to assess the reliability of the information obtained by these computational studies, molecular docking studies also included: (i) two FCG highly related analogues AAT 9d and AAT **10d**, featuring F508del CFTR corrector ability [36], and (ii) a reference corrector known as **corr4a** and the conformationally locked derivatives **9e** and 10c, exhibiting a seven- and eight-membered ring, respectively, as constrained bithiazoles (see the chemical structures in Figure 6) [48,49,50].

Notably, both series of correctors (AATs and **corr4a** congeners) experienced several lipophilic substitutions aimed at improving their potency, and also endowed with comparable F508del CFTR corrector ability. Along with this, biological studies previously reported in literature disclosed **corr4a**, as well as the constrained analogues, as NBD2-targeting CFTR modulators [27].

**Figure 7:**
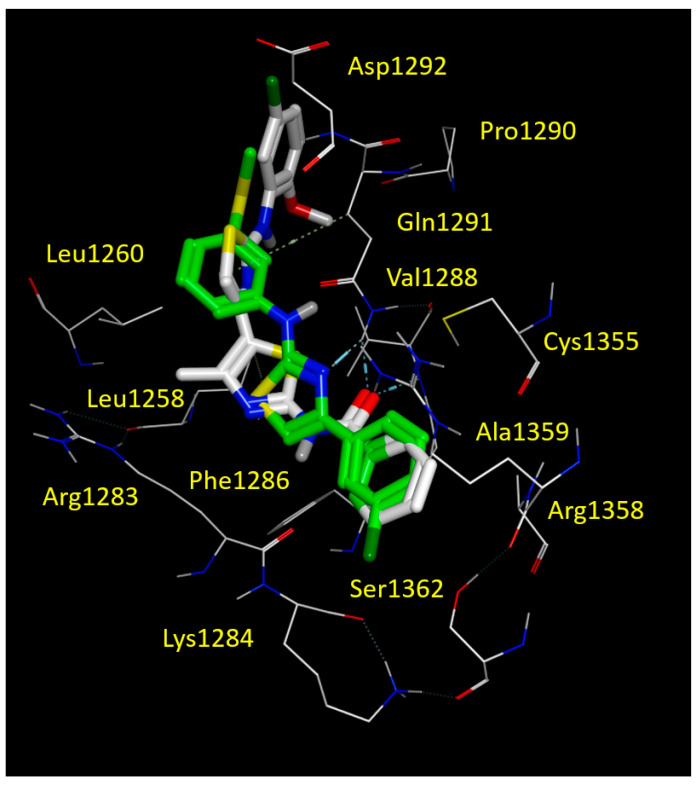
Docking positioning of corrector **corr4a** (C atom; white) and FCG (C atom; green) at the CFTR NBD2 domain (PDB code = 6UK1). All residues placed 3.5 Å from the ligands are shown. Hydrophobic, polar, negatively and positively charged amino acids are shown in green, pink, and by red and blue circled pink labels, respectively.

We found that FCG was highly stabilized at the NBD2 cavity due to hydrophobic contacts involving the 3-SCH_3_-phenyl ring and Leu1255, Leu1258 and Leu1260, while the terminal 3-Cl-phenyl group was engaged in Van der Waals interactions with Cys1355, Ala1359 and cation-π contacts with Arg1358 (Figure 7 and Figure S2A).

This kind of positioning also allowed the compound to be H-bonded to Gln1292 by means of the nitrogen atom of the thiazole core. It should be noticed that the reference **corr4a** maintained the same docking mode, i.e., the oxygen atom of the carbonyl group engaged in H-bonds with the Gln1291 and Val1288. On the other hand, the benzoyl group of **corr4a** highly mimicked the Cl-phenyl motif of FCG, having comparable interactions with Cys1355, Arg1358 and Ala1359 (see Figure 7 and Figure S2B). Furthermore, the amino-thiazole core and the di-substituted phenyl ring of **corr4a** phenyl rings proved to be superposed on the 3-SCH_3_-phenyl group of FCG, and surrounded by Leu1255, Leu1258 and Leu1260. In this way, **corr4a** displayed the same hydrophobic contacts with the leucine residue of the NBD2 binding site, suggesting a key role played by hydrophobic substituents linked to a central H-bonding core (see Figure S2B).

**Figure 8:**
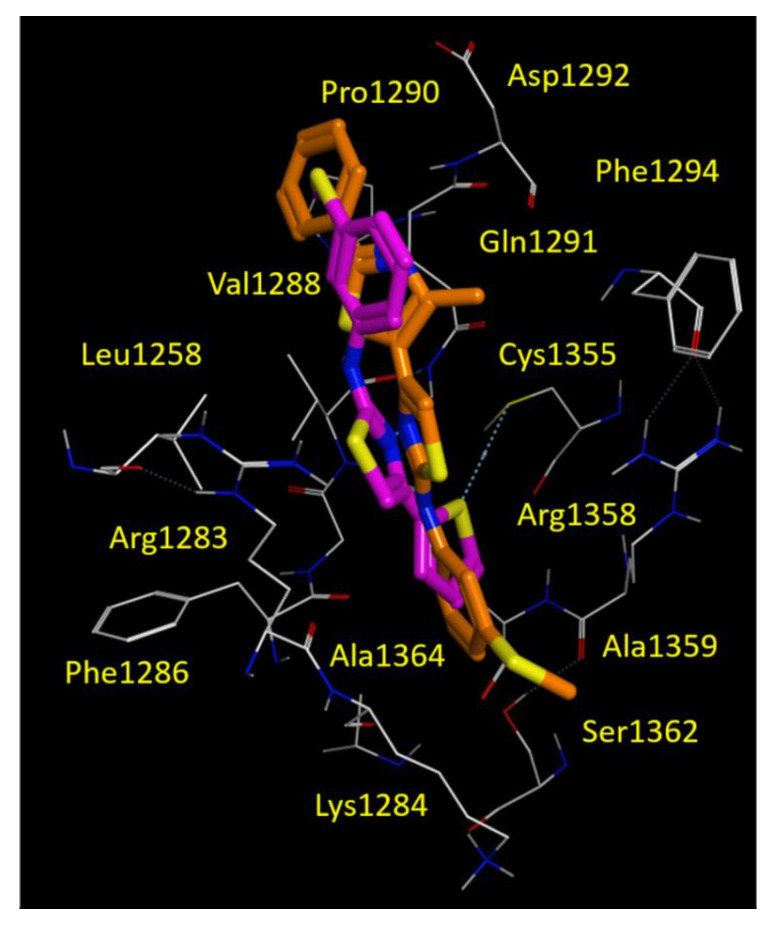
Docking positioning of the AAT **9d** (C atom; magenta) and **10d** (C atom; orange) at the CFTR NBD2 domain (PDB code = 6UK1). All residues placed 3.5 Å from the ligands are shown. Hydrophobic, polar, negatively and positively charged amino acids are shown in green, pink, and by red and blue circled pink labels, respectively.

Accordingly, within the AAT series, the **10d** corrector also moved the phenyl-substituted thiazole portion in proximity of the 3-SCH_3_-phenyl ring of FCG, as we previously described for **corr4a**. Conversely, the SCH3-phenyl group of **10d** was projected towards Cys1355, Ala1359 and with Arg1358, detecting Van der Waals and cation-π contacts, respectively (see Figure 8).

The thiophene containing **9d** proved to be an effective bioisostere of FCG, maintaining the thiophene ring and the 3-SCH3-phenyl one near the 3-Cl-phenyl and the 3-SCH3-phenyl rings of FCG (see Figure 8). Both correctors **9d** and **10d** maintained the previously cited H-bond with Gln1291, due to the nitrogen atom of the main thiazole core (Figure 8 and Figure S3).

Regarding the **corr4a** analogues **9e** and **10c**, the two constrained thiazoles experienced similar docking positioning, having the proper H-bonds with Gln1291 by means of the nitrogen atom of the thiazole group bearing the carboxamide moiety (see Figure S4). In particular, the presence of the seven- or eight-membered ring in **9e** and **10c** moved the corrector amino-phenyl group in the deep cavity of the NBD2 domain including Phe1286, Phe1294, Phe1296 and Pro1306 displaying π-π stacking and hydrophobic contacts. However, Van der Waals interactions involving the terminal substituent linked to the carboxamide group and Leu1254, Leu1255, Leu1258 were maintained.

### 3.8. In Silico Evaluation of ADME Properties

Using computational methods, we analysed the putative absorption, distribution, metabolism, excretion (ADME) profile of FCG, its analogues **9d** and **10d**, as well as of the reference thiazole-containing CFTR modulators **corr4a**, **9e** and **10c** [48,50]. Thus, the molecular weight (MW), logarithmic ratio of the octanol-water partitioning coefficient (cLogP), the number of rotatable bonds (nRB), number of H-bond acceptor atoms (nHBA) and donor atoms (nHBD) and the topological polar surface area (TPSA) were evaluated in silico. In addition, absorption at the human intestinal level (HIA), volume of distribution (Vd), the plasmatic protein binding event (%PPB) and compound affinity with respect to human serum albumin (LogKa^HSA^) were calculated to explore the putative value of the oral bioavailability as a percentage (%F).

As shown in Table 1, all the compounds were predicted to be endowed with high human intestinal absorption, being FCG (and **9d**) characterized by better lipophilicity and logP values than **corr4a**, indicating that FCG could be proposed as lead compound for the multi-drug treatment of the basic defect underlying CF [51,52].

## 4. Discussion

Among CF causing mutations, F508del CFTR is the most common, being found in 80% to 90% of CF patients [53]. It belongs to CFTR class II mutations, which lead to protein misfolding and aberrant trafficking to the plasma membrane, resulting in a lack of CFTR functional expression on the cell surface. Small chemicals, called correctors, initially discovered by high throughput screening [21,22,27,31,54] are being continuously developed and tested for their capability to improve CFTR folding and assembly, and enhance CFTR trafficking and expression on the plasma membrane.

Research efforts focused on understanding corrector mechanism of action have demonstrated that correction provided by pharmacological chaperones, molecules that facilitate F508del CFTR folding by putative direct binding to the mutant CFTR, is generally higher than that exerted by proteostasis regulators acting as modulators of CFTR synthesis, folding and degradation pathways [55,56]. Nevertheless, the paucity of clinical outcomes achieved by treatment with the first approved single corrector [24,57] has pushed toward the search for more efficient CF therapies. In short, studies aimed at evaluating the potential of combinations of correctors provided evidence that the use of combinations of drugs addressed to target distinct sites of F508del CFTR, or to rescue different structural and functional defects or different steps of its folding pathway, could provide the necessary correction to attain the rescue benefits required clinically [27,28,29,31,58]. This concept has already produced a combination of two correctors (plus a potentiator) actually approved for the clinical use in patients [59].

In this study, we focused our attention on the mechanism of action of two molecules, FCG and VX809, known to rescue F508del CFTR functional expression when used individually [36,37,38,39,40], showing that their use in combination can provide a synergistic improvement of mutant CFTR expression. Furthermore, by exploiting the properties of a heterologous expression system constituted by the highly transfectable HEK-t cell line, we expressed different CFTR domains and groups of domains and demonstrated that the synergistic effect exerted by the two molecules is due to their interaction with different domains of the F508del CFTR protein.

As first, we ascertained whether FCG and VX809, administrated individually or in combination, could influence cell viability or CFTR construct transfection efficiency. In accordance with previous results obtained for VX809 [29], the trypan blue exclusion test provided low toxicity values for both compounds. This result allowed us to use micromolar concentrations of both compounds to be assayed for their efficacy in the rescue of CFTR expression. The quantitative assessment of the mRNAs coding for CFTR constructs by real-time PCR confirmed that the expression of the transcripts encoding WT and F508del CFTR, M1, WT and F508del N1, M2, N2, WT and F508WT M1N1 and WT and F508del ∆NBD2 (see Supplementary Tables S2–S4) were comparable in all preparations, independently from the treatment (or not) with the molecules under study. These results allow us to conclude that both FCG and VX809 exert their effect on CFTR expression post-transcriptionally.

We then tested whether FCG and VX809 show the same effect on the expression level of WT and mutant F508del CFTR. To achieve this aim, we compared the expression level of total CFTR protein (B + C band) as well as its mature form (C/(C + B) band ratio) in SDS-PAGE whole cell lysates obtained from vehicle DMSO and compound-treated samples. Our analysis showed that in WT CFTR lysates, neither total nor mature WT CFTR protein increased after treatment with FCG and VX809 administrated individually or in combination (Figure 1A). On the contrary, total F508del CFTR protein expression level significantly increased after incubation with the two compounds administrated individually or in combination. Our results also point out that FCG alone was not able to fully restore the processing of F508del CFTR, as evidenced by the lack of the increase of the C/(C + B) band ratio, the opposite occurs when VX809 is applied alone or in combination with FCG. It is that the best level of F508del CFTR maturation was observed with the FCG + VX809 combination (Figure 1B), confirming that the two molecules act in synergy, providing together a higher correction than each compound alone. Furthermore, as already noticed for VX809 and other correctors [36,37,38,39,40], FCG and VX809 seem to be able to discriminate between the WT and the F508del isoforms, interacting only with those regions of the F508del CFTR whose folding or assembling are different from that of WT CFTR [38,39,40] or, alternatively, modulating any component of the cellular machinery responsible for defective CFTR protein degradation, preventing the mutant CFTR from premature disruption by the endoplasmic reticulum associated protein degradation system (ERAD), or even inducing the endoplasmic reticulum (ER) quality control complex to recognize it as a protein that is prone to leave the ER compartment.

Another aspect developed in this study concerns the identification of mutant CFTR regions that are mainly affected by the action of FCG. To achieve this goal, we generated expression constructs containing CFTR single domains: MSD1 (M1, residues 1–388), NBD1 (N1, residues 348–633) in both WT and F508del isoforms, R (residues 645–834), MSD2 (M2, residues 837–1218) and NBD2 (N2, residues 1210–1480) [40].

As highlighted by Figure 2, FCG and VX809 were able to increase the expression of MSD1 and NBD2, respectively, while they had an almost negligible effect on the expression level of the other CFTR domains, either when administrated individually or in combination. As FCG contains a thiazole scaffold, the results are not surprising, since other compounds with a similar chemical structure such as **corr4a**, have been proposed to bind and interact with NBD2 [25,40,60]. Regarding VX809, even if there is still debate on its precise binding site on CFTR [61], our findings are in agreement with those of other research groups that identified the MSD1 as the target of its action in terms either of direct binding and domain stabilization [38,39,40,62].

Successively, we verified whether the findings obtained with isolated CFTR domains were confirmed when FCG and VX809 compounds were administrated alone on in combination to HEK-t cells expressing larger portions of the CFTR protein. To achieve this, we generated constructs encoding WT and F508del CFTR N-half (M1N1, residues 1–533), CFTR C-half (M2N2, residues 837–1480) and WT and F508del CFTR lacking the NBD2 domain (ΔNBD2, residues 1–1172) and transiently transfected them into HEK-t cells.

In agreement with the results obtained with isolated CFTR domains, for both WT and F508del isoforms FCG failed to enhance the expression level of CFTR N-half (Figure 3A,B). On the contrary, VX809 increased the expression level of both isoforms of this region. The increase of the expression level determined by the combination of the two drugs was comparable to that exerted by VX809 alone, confirming that the first half of the CFTR protein is not necessary to FCG to exert its action.

Then, we performed analogous experiments with the M2N2 segment. Even if this polypeptide fails to express the fully glycosylated form when transfected without its counterpart, in the CFTR C-half [37,38,40], in SDS-PAGE lysates from M2N2 transfected HEK-t cells treated with either FCG and FCG + VX809, we noticed a significant increase of M2N2 expression (core-glycosylated + unglycosylated isoforms) with respect to control DMSO treated samples (Figure 3C). On the contrary, VX809 did not influence the total expression level of this CFTR half. In agreement with what we observed with the F508del CFTR whole structure, FCG administrated alone or in combination with VX809 did not promote the transition from the unglycosylated to the core-glycosylated form of the M2N2 polypeptide.

This finding allowed us to hypothesize that the FCG molecule would need a further modification to improve its efficacy as a CFTR corrector or, alternatively, that a third molecule, a modulator specifically addressed to exert its effect along the CFTR maturation pathway, would be useful to boost the effect of the FCG and VX809 combination. Indeed, the use of a triple combination of drugs to improve CFTR functional expression is a well-accepted concept for the treatment of CF. As an example, Trikafta, the combination therapy combining two correctors (i.e., Elexacaftor + Tezacaftor) and the potentiator ivacaftor, have been recently approved by FDA and EMA for the treatment of CF in patients aged 6 years and older who have at least one copy of the F508del mutation [59,63].

To definitively confirm that within F508del CFTR the NBD2 domain is effectively the target of FCG, we compared the expression level of ΔNBD2, a CFTR construct that lacks this domain [40], before and after treatment with FCG or VX809, administrated singly or in combination. As shown in Figure 4, this construct maintains the capability of expressing both bands B and C of the CFTR bands corresponding to the mature, fully glycosylated (B band), and to the immature core glycosylated forms of the CFTR protein (C band), respectively [40,64]. Analogously to what we observed with the full-length WT CFTR, both FCG and VX809, administrated singly or in combination, did not have any effect on the expression of WT ΔNBD2 polypeptide (Figure 4A). Treatment with FCG was ineffective in rescuing the folding and trafficking defects of the F508del ∆NBD2 (lane 2 of Figure 4B). On the contrary, treatment with VX809 and FCG + VX809 caused a significant increase of the level of total protein (B + C band) and mature protein (C/(C + B) band) fractions of F508del ∆NBD2. The enhancement of F508del ∆NBD2 expression level caused by the concomitant use of FCG and VX809 was comparable to that of VX809, confirming once more that NBD2 is necessary to FCG to exert its effect.

To further strengthen that the NBD2 is the region whose expression is mostly affected by FCG, we performed an analysis of the stability of this domain using a cycloheximide chase assay. We hypothesized that a possible mechanism of action of FCG in enhancing NBD2 expression could be linked to its capability in slowing the turnover rate of this domain. To verify this hypothesis, we transfected HEK-t cells with NBD2 and incubated them in the presence of FCG, VX809, FCG + VX809 or DMSO. The next day, cycloheximide was added to inhibit protein synthesis. Whole cell extracts were collected at various time points and subjected to immunoblot analysis. As expected, FCG enhanced the stability of NBD2 (Figure 5B) while VX809 failed to exert this kind of action (Figure 5C). The combination FCG + VX809 promoted an increase of NBD2 stability that was similar to that of FCG alone (Figure 5D).

However, an open question remains. We observed an increase in the expression of M1 and N2 as a result of treatment with VX809 and FCG, respectively. Clearly, neither domain contains the F508del mutation, but unlike WT CFTR they respond to the correctors. This paradox becomes more evident when examining the results obtained with M1N1 WT and M2N2. Although, at the moment, we do not have data that can explain this contradiction, we hypothesize that the correct folding of WT CFTR could “hide” the regions of the protein involved in the action of the correctors. Obviously, a more detailed analysis is necessary to solve this puzzling question.

To corroborate our findings, we performed a computational analysis aimed at exploring the putative FCG binding domain on NBD2. Our in silico studies highlighted a binding pocket for FCG and for other thiazole-containing correctors, such as **corr4a**, promoting hydrophobic contacts with Leu1255, Leu1258 and Leu1260. In addition, the binding ability of the compounds to this pocket was favoured by H-bonding to Gln1291. Along with this, further π-π stacking and cation-π contacts with Arg1358 proved to be relevant in NBD2-targeting, as previously discussed for the constrained thiazoles **9e** and **10c**. The PK profile of FCG proved to be endowed by more promising logP and bio-availability properties, highlighting once more that the chemo-type of AATs deserves to be further exploited for the discovery of effective CFTR modulators.

Finally, it is worth noting that new molecules showing the ability to rescue the basic F508del CFTR defect often show discordant efficacy when tested in different cell systems, including primary cultures of bronchial epithelial cells [65,66]. In fact, it is possible that the contribution of the cell machineries that control maturation, trafficking, and degradation of CFTR is different from a cell model to another. Consequently, the F508del-CFTR pharmacological rescue of a given compound may be different when it is tested in different cell types. As a consequence, the efficacy of FCG, as well as of new and more effective ATTs derivatives, should be also validated on epithelial cell models that have the characteristics of CF- affected human airways such as CFBE or 16HBE.

In summary, three major conclusions can be derived from the results of this study. First, our analysis confirms that FCG, as well as VX809 and other correctors [38,39,40], show different behaviour towards full-length WT and F508del CFTR molecules, increasing the expression of only the mutant CFTR isoform. Second, although we have not provided any direct evidence that correctors directly bind to the F508del CFTR, our findings indicate that FCG and VX809 specifically and positively influence the expression and the stability of two different regions of F508del CFTR: NBD2 and TMD1, respectively. Third, our results reinforce, once more, the concept that the use of drug combinations is the best choice for the correction of the multiple defects that affect F508del CFTR. Given the wide variety of different drug combinations, and the possibility to improve the chemical properties and structures of already tested lead compounds, it is realistic to think of the development of personalized combinations of CFTR modulators as a realistic option for the treatment of patients bearing class II and III mutations.

## Figures and Tables

**Table 1:** Calculated ADME descriptors related to absorption and distribution properties of FGC, corr 4a and their congeners.

Molecule	FCG	9d	10d	Corr4a	9e	10c
cLogP	5.34	4.79	5.6	5.42	5.2	6.5
MW	332.87	304.46	395.57	456.97	493	463.02
TPSA	78.46	106.7	119.59	132.62	158.92	132.62
nHBA	2	2	3	6	8	6
nHBD	1	1	1	2	2	2
nRB	4	4	5	6	8	5
HIA (%)	100	100	100	100	100	100
Vd (l/kg)	3.8	2.5	4.6	3.8	3.6	4.4
%PPB	99.7	99.2	99.7	99.8	98.2	99.3
LogKa HSA	5.05	4.77	4.84	5.03	3.75	4.23
%F (oral)	69	96.2	73.8	64.8	87.4	21

## Data Availability

The data presented in this study are available in the article and Supplementary Material.

## Supplementary Materials

The following are available online at https://www.mdpi.com/article/10.3390/biom11101417/s1, Figure S1: Constructs used in the study, Table S1: Real-time PCR conditions, Table S2: Raw data of cell viability, Tables S3–S5: Raw data of real time PCR experiments, Table S6: Raw data of protein expression in cycloheximide chase assays, Figures S2 and S3: Supporting figures of molecular docking analyses.
