# Supplementary material for: NBD2 Is Required for the Rescue of Mutant F508del CFTR by a Thiazole-Based Molecule: A Class II Corrector for the Multi-Drug Therapy of Cystic Fibrosis
Source: Biomolecules. 2021 Sep 28;11(10):1417. doi: 10.3390/biom11101417 (PMC8533355; doi:10.3390/biom11101417)
Supplement: Supplementary file 1 [file biomolecules-11-01417-s001.zip › biomolecules-1337965-supplementary.pdf]

NBD2 Is Required for the Rescue of Mutant F508del CFTR by a Thiazole-Based Molecule: A Class II Corrector for the Multi-Drug Therapy of Cystic Fibrosis

Chiara Brandas [1], Alessandra Ludovico [1], Alice Parodi [2], Oscar Moran [1], Enrico Millo [2], Elena Cichero [3] and Debora Baroni [1,*]

[1] Istituto di Biofisica, Consiglio Nazionale delle Ricerche, Via De Marini 6, 16149 Genova, Italy; brandas.chiara@hsr.it (C.B.); alessandra.ludovico@ibf.cnr.it (A.L.); oscar.moran@ibf.cnr.it (O.M.)

[2] Department of Experimental Medicine, Section of Biochemistry, University of Genova, Viale Benedetto XV 1, 16132 Genova, Italy; alice.parodi1994@gmail.com (A.P.); enrico.millo@unige.it (E.M.)

[3] Department of Pharmacy, Section of Medicinal Chemistry, School of Medical and Pharmaceutical Sciences, University of Genova, Viale Benedetto XV, 3, 16132 Genova, Italy; cichero@difar.unige.it

* Correspondence: debora.baroni@ibf.cnr.it

Supplementary materials

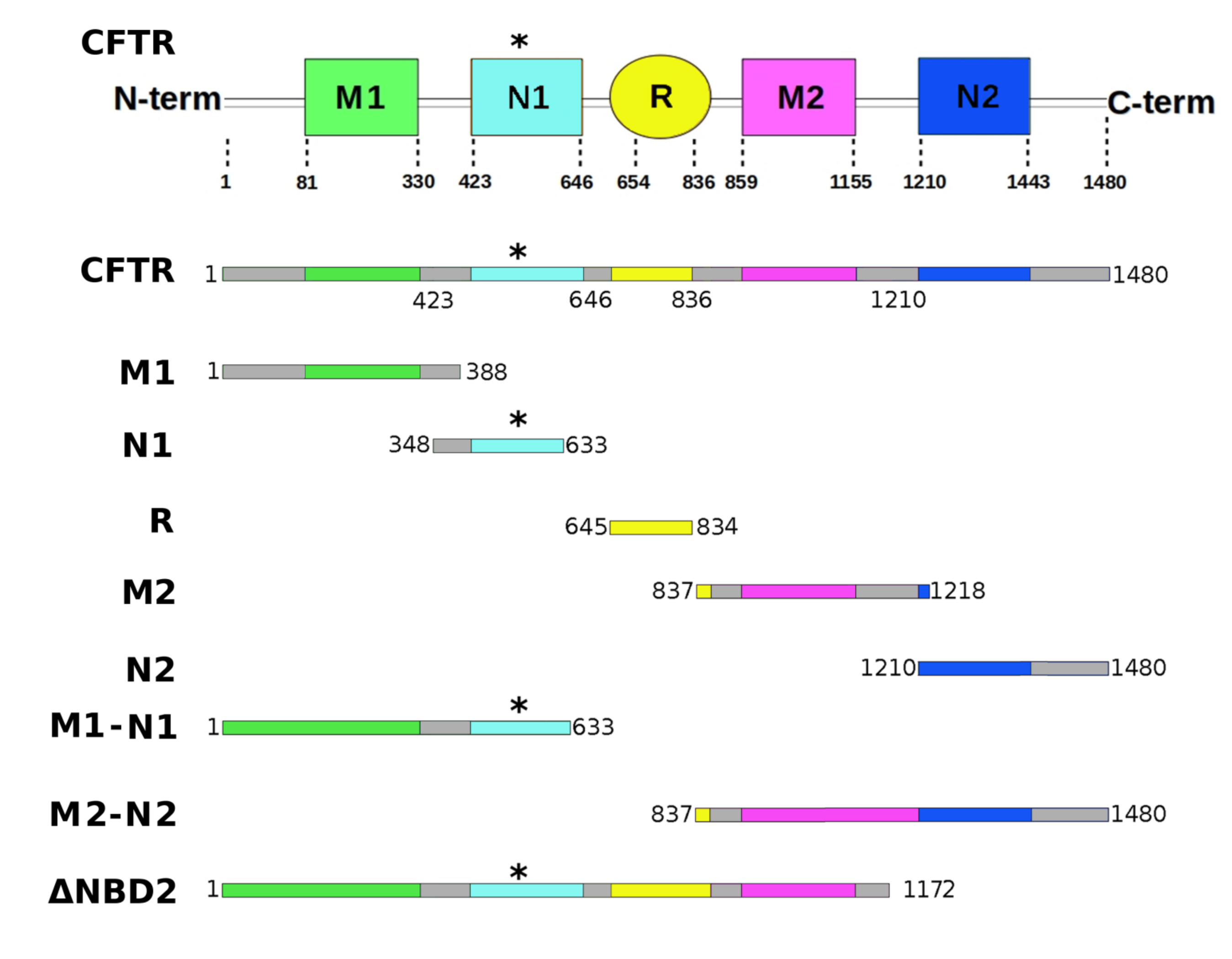

Figure S1. cDNA constructs used in this work. M1(transmembrane domain 1), N1 (nucleotide binding domain 1), R (regulatory domain), M2 (transmembrane domain 2) and N2 (nucleotide binding domain 2) domains are depicted with red, cyan, magenta and blue boxes, respectively. The gray boxes indicate the inter-domain regions. The first and the last residue of the sequence of each construct is also shown. Asterisks indicate those cDNAs encompassing the phenylalanine at position 508 of the CFTR molecule that have been produced and used in both isoforms.

Table S1. Sequences of forward and reverse primers used to amplify WT and F508del CFTR, M1, WT and F508del N1, R, M2, N2, WT- and F508del-M1N1, M2N2, and WT and F508del ΔNBD2 constructs and the size expected for each PCR-amplified product. The sequence to amplify the "housekeeping" gene glyceraldehyde-3-phosphate-dehydrogenase (GAPDH) is also shown.

Target sequence	Forward Primer	Reverse Primer	Amplicon size (bp)
M1<br>WT/F508del-M1N1;<br>WT/F508del-CFTR;<br>WT/F508del-ΔNBD2	5'-GCCAGCGTTGTCTCCAAAC-3'	5'-GCGTTCCTCCTTGTTATCC -3'	327
WT/F508del N1	5'- CACCACCATCTCATTCTGC-3'	5'- CAACCGCCAACAACTGTCC-3'	359
N2<br>WT/F508del-M2N2	5'-GAACAGTTTCCTGGGAAGC-3'	5'-CTAAAGCCTTGTATCTTGC-3'	459
R	5'-TCTACAGCCAGACTTTAGCT-3'	5'- GTGCTGATCACGCTGATGC -3'	366
M2	5'-CAGTGATTATCACCAGCACC-3'	5'- CAGGATAATACCAACTCTTCC -3'	689
GAPDH	5'-CAAGGTCATCCATGACAACTTTG-3'	5'-GTCCACCACCCTGTTGCTGTAG-3'	496

Reaction mixtures were processed in a CFX Connect Real-Time PCR Detection System instrument (Bio-Rad Laboratories, Hercules, CA, USA). The PCR reaction mixture contained 1.5 µM of the appropriate forward and reverse PCR primers (Bio-Fab Research, Roma, Italy), 1.5 U of SsoAdvance Universal SYBR Green Supermix (Bio-Rad Laboratories) and 100 ng cDNA in 20 µl. The PCR protocol consisted of an initial denaturation at 95 °C for 30 s, followed by 35 cycles of a denaturation step at 95 °C for 10 s, an annealing step at 54 or 56 °C for 30 s and an extension step at 72 °C for 50 s. Appropriate negative and positive controls were included. A melting curve analysis was used to evaluate the presence of secondary non-specific products.

Table S2. Viability of HEK293 cells after 24 hour exposition to different concentrations of FCG and VX809, as evaluated by the Trypan blue exclusion method.

**FCG concentration (μM)**	**Survival rate (%) (mean ± sem)**		**VX809 concentration (μM)**	**Survival rate (%) (mean ± sem)**
50	50.3 ± 0.6		50	55.8 ± 3.4
30	64.5 ± 3.5		32	59.6 ± 6.3
25	68.7 ± 2.1			
20	71.2 ± 2.7			
15	78.3 ± 3.4		16	62.8 ± 0.9
10	85.1 ±0.5			
8	87.1 ± 2.0		8	70.5 ± 3.0
4	92.8 ± 0.5		4	76.6 ± 1.3
2	95.9 ± 0.3		2	82.5 ± 1.4
1	97.1 ± 0.7		1	89.2 ± 0.2
0.5	98.3 ± 0.3		0.5	92.8 ± 0.8
0.25	98.7 ± 0.2		0.25	94.7 ± 0.9
			0.125	99.7 ± 0.9
0 (DMSO)	99.9 ± 0.1		0 (DMSO)	99.8 ± 0.1

Table S3. Evaluation of CFTR mRNA abundance in HEK-t cells transfected with WT- or F508del-CFTR after treatment with FCG, VX809, and FCG + VX809. The mRNA abundance is expressed as the cycle threshold (Ct) obtained by real-time PCR and normalized to the expression of the GAPDH used as housekeeping gene. Data represent the mean±sem (standard error of the mean). For each condition, the number of observations is indicated into the brackets. Comparisons of values with those of control (untreated) cells were made with the Dunnet test. Probability p values are reported. Differences are significant when $p < 0.05$.

**CFTR Constructs**	**Control**	**FCG**		**VX809**		**FCG+VX809**	
	**Ct**	**Ct**	**p**	**Ct**	**p**	**Ct**	**p**
**WT**	0.93 ± 0.07 (6)	1.01 ± 0.07 (5)	> 0.99	1.00 ± 0.06 (7)	> 0.99	0.93 ± 0.09 (6)	0.83
**F508del**	0.83 ± 0.08 (6)	0.91 ± 0.06 (6)	0.93	0.90 ± 0.13 (6)	0.96	0.99 ± 0.27 (3)	> 0.99

Table S4. Evaluation of mRNA abundance in HEK-t cells transfected with M1, WT or F508del N1, R, M2 and N2 after treatment with FCG, VX809, and FCG + VX809. The mRNA abundance is expressed as the cycle threshold (Ct) obtained by real-time PCR and normalized to the expression of the GAPDH used as housekeeping gene. Data represent the mean ± sem (standard error of the mean). For each condition, the number of observations is indicated into the brackets. Comparisons of values with those of control (untreated) cells were made with the Dunnet test. Probability p values are reported. Differences are significant when $p < 0.05$.

Construct	Control	FCG		VX809		FCG + VX809	
	Ct	Ct	p	Ct	p	Ct	p
**M1**	1.08 ± 0.05 (5)	0.97 ± 0.01 (4)	0.10	0.89 ± 0.05 (3)	0.29	0.88 ± 0.04 (3)	0.49
**WT-N1**	0.98 ± 0.09 (3)	0.97 ± 0.08 (3)	0.85	1.10 ± 0.12 (3)	> 0.99	0.97 ± 0.07 (3)	> 0.99
**F508del-N1**	0.97 ± 0.03 (3)	1.05 ± 0.04 (3)	0.32	0.98 ± 0.05 (3)	0.38	0.90 ± 0.03 (3)	0.68
**R**	0.95 ± 0.13 (4)	0.91 ± 0.07 (5)	0.92	0.99 ± 0.13 (5)	> 0.90	0.94 ± 0.09 (3)	> 0.90
**M2**	1.11 ± 0.10 (3)	1.10 ± 0.14 (3)	> 0.99	1.25 ± 0.08 (5)	0.47	1.29 ± 0.07 (5)	0.42
**N2**	1.08 ± 0.11 (3)	1.05 ± 0.07 (3)	0.89	1.12 ± 0.07 (3)	> 0.99	1.25 ± 0.13 (3)	0.88

Table S5. Evaluation of mRNA abundance in HEK-t cells transfected with WT or F508del M1N1, M2N2 or WT- or F508del- ΔNBD2 after treatment with FCG, VX809, and FCG + VX809. The mRNA abundance is expressed as the cycle threshold (Ct) obtained by real-time PCR and normalized to the expression of the GAPDH used as housekeeping gene. Data represent the mean ± sem (standard error of the mean). For each condition, the number of observations is indicated into the brackets. Comparisons of values with those of control (untreated) cells were made with the Dunnet test. Probability p values are reported. Differences are significant when $p < 0.05$.

Construct	Control	FCG		VX809		FCG + VX809	
	Ct	Ct	p	Ct	p	Ct	p
WT-M1N1	0.98 ± 0.07 (5)	1.05 ± 0.01 (3)	0.83	1.01 ± 0.05 (3)	0.88	0.85 ± 0.04 (3)	> 0.99
F508del-M1N1	1.00 ± 0.18 (3)	1.06 ± 0.23 (4)	> 0.99	1.16 ± 0.06 (5)	> 0.53	1.05 ± 0.01 (3)	> 0.56
M2N2	1.05 ± 0.01 (6)	1.03 ± 0.05 (6)	0.08	0.92 ± 0.05 (6)	0.52	0.98 ± 0.08 (8)	0.85
WT-ΔNBD2	1.08 ± 0.05 (3)	1.18 ± 0.03 (3)	> 0.99	1.20 ± 0.04 (3)	0.36	1.05 ± 0.06 (3)	0.13
F508del-ΔNBD2	1.03 ± 0.01 (3)	1.06 ± 0.02 (3)	0.28	0.96 ± 0.02 (3)	> 0.99	1.15 ± 0.01 (3)	0.74

Table S6. Evaluation of M2 protein expression in HEK-t cells whole cell lysates after treatment with correctors and protein new synthesis blockage with cycloheximide. Quantification of protein expression was obtained analyzing the band intensity with the software imageJ. Retrieved values were normalized to the intensity of the protein actin used as housekeeper protein and to the value of M2 protein expression at time 0. Data represent the mean ± sem (standard error of the mean). For each condition, samples were run in triplicate. Comparisons of values with those of control, untreated samples were made with the Dunnet test. Probability p values are reported. Differences are significant when $p < 0.05$.

Time	Control	FCG		VX809		FCG+VX809	
	Protein expression	Protein expression	P	Protein expression	P	Protein expression	P
0 hour	1.00 ± 0.05	1.00 ± 0.02	0.77	1.00 ± 0.03	0.55	1.00 ± 0.03	0.27
1 hour	0.90 ± 0.04	0.93 ± 0.03	0.54	0.92 ± 0.03	0.84	0.96 ± 0.07	0.19
2 hours	0.73 ± 0.05	0.83 ± 0.02	P<0.01 (*)	0.78 ± 0.04	0.71	0.86 ± 0.04	P<0.01 (*)
4 hours	0.43± 0.06	0.58 ± 0.05	P<0.01 (*)	0.45 ± 0.06	p>0.99	0.61 ± 0.03	P<0.01 (*)
6 hours	0.35 ± 0.05	0.46± 0.02	P<0.01 (*)	0.32 ± 0.05	0.87	0.47 ± 0.04	P<0.01 (*)
8 hours	0.31 ± 0.04	0.34 ± 0.03	0.78	0.29 ± 0.06	0.77	0.32 ± 0.05	0.78

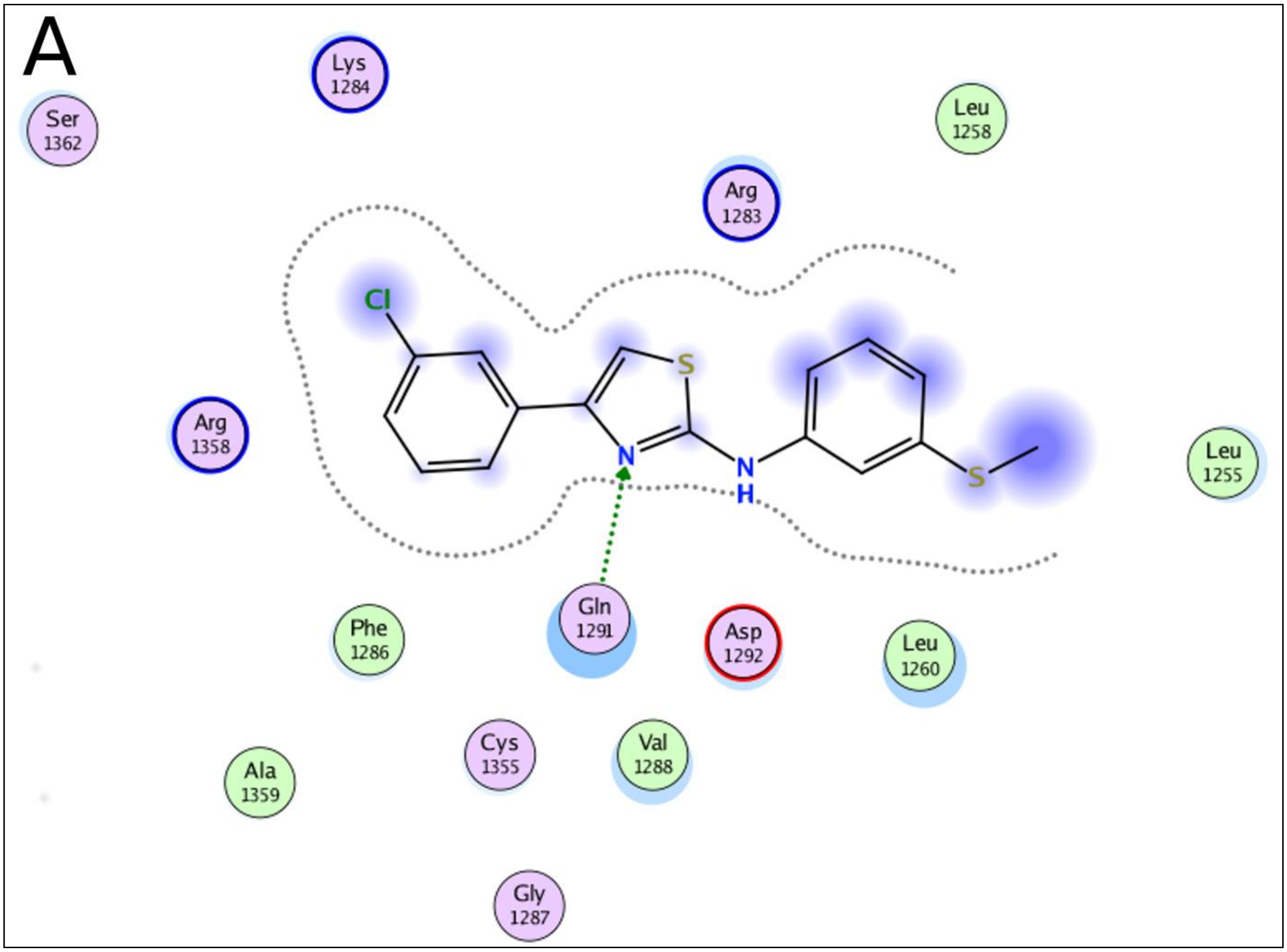
A
Lys 1284
Ser 1362
Leu 1258
Arg 1283
Cl
S
Arg 1358
N
N
H
S
Leu 1255
Gln 1291
Phe 1286
Asp 1292
Leu 1260
Cys 1355
Val 1288
Ala 1359
Gly 1287

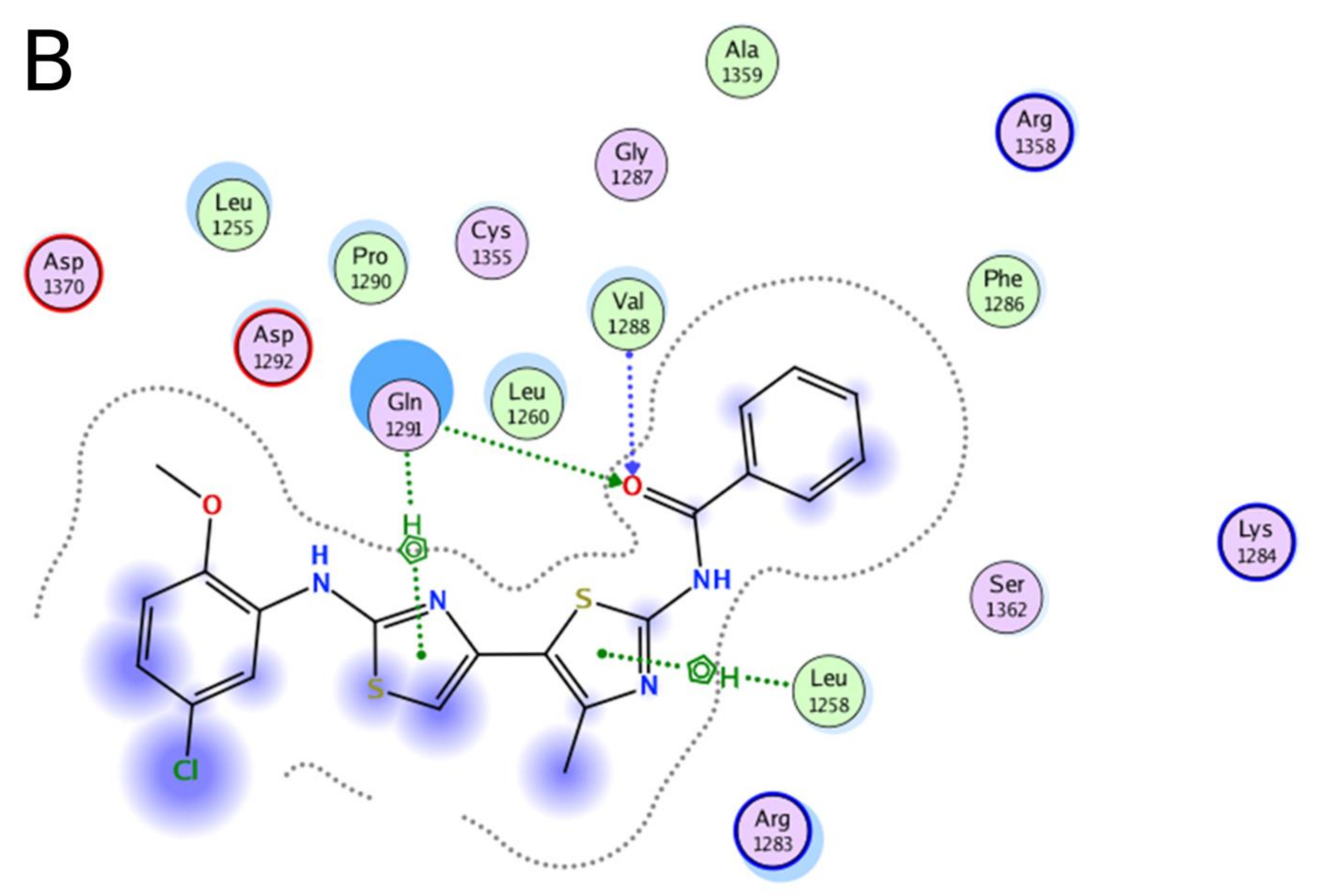

Figure S2. Docking positioning (ligplot) of FCG (A) and corr4a (B) at the CFTR NBD2 domain (PDB code = 6UK1). All those residues placed 3.5 Å from the ligands are shown. Hydrophobic, polar, negatively- and positively- charged amino acids are shown in green, pink, and by red and blue circled pink labels, respectively.

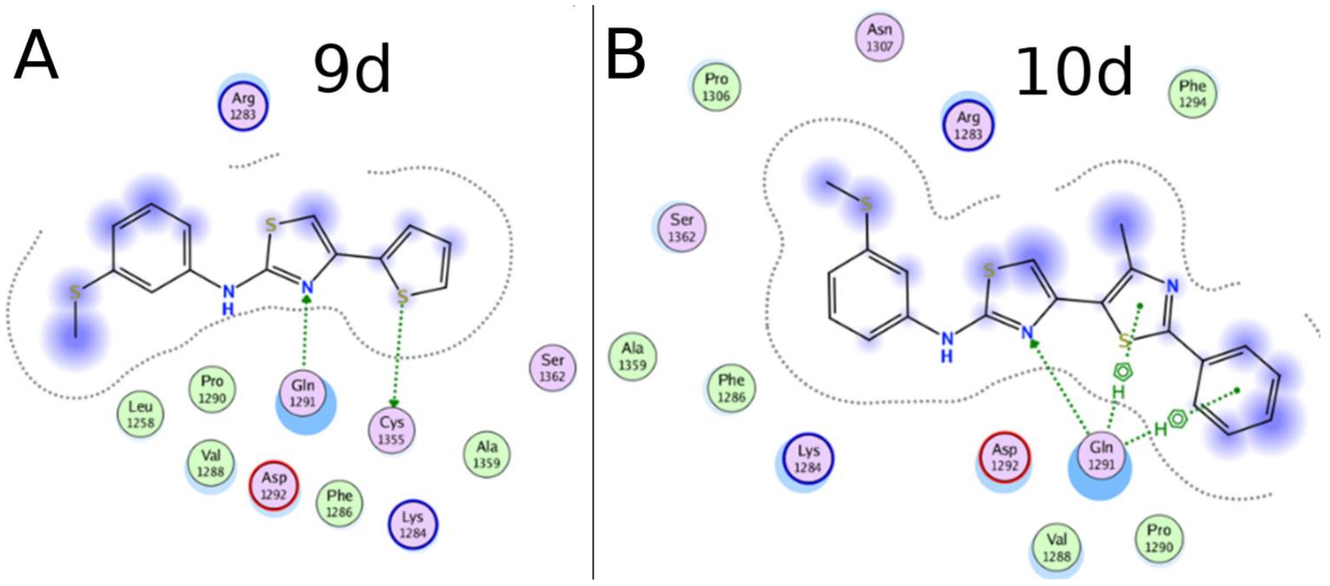

Figure S3. Docking positioning of correctors 9d and 10d at the CFTR NBD2 domain (PDB code = 6UK1). All those residues placed 3.5 Å from the ligands are shown. Hydrophobic, polar, negatively- and positively- charged amino acids are shown in green, pink, and by red and blue circled pink labels, respectively.

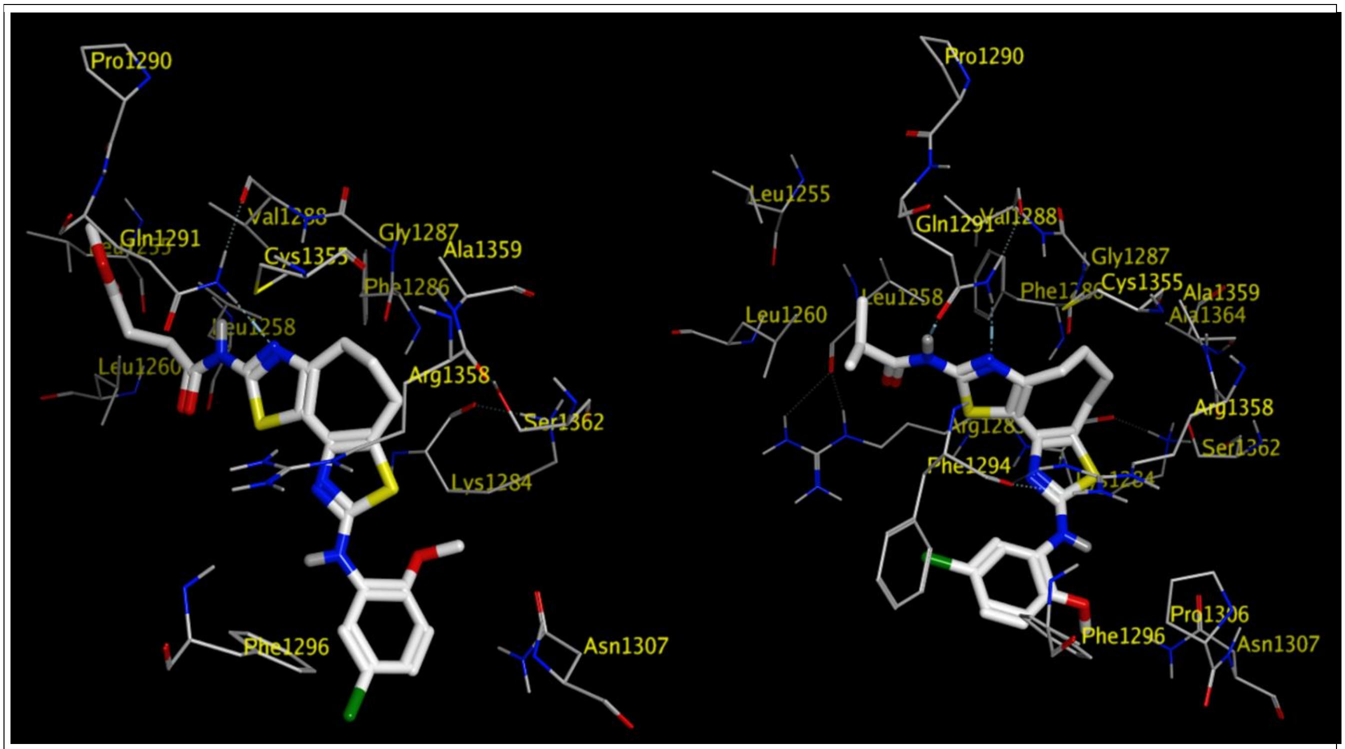

Figure S4. Docking positioning of the two bithiazole correctors **9e** (C atom; white) and **10c** (C atom;

white) at the CFTR NBD2 domain (PDB code = 6UK1). All those residues placed 3.5 Å from the ligands are shown. Hydrophobic, polar, negatively- and positively- charged amino acids are shown in green, pink, and by red and blue circled pink labels, respectively.
